# Race, Discharge Disposition, and Readmissions After Elective Hip Replacement: Analysis of a Large Regional Dataset

**DOI:** 10.1089/heq.2019.0083

**Published:** 2019-12-16

**Authors:** Bella Mehta, Jasvinder A. Singh, Kaylee Ho, Michael Parks, Charles Nelson, Debra D'Angelo, Said A. Ibrahim

**Affiliations:** ^1^ Department of Rheumatology, Hospital for Special Surgery, New York, New York.; ^2^ Department of Rheumatology, Weill Cornell Medicine, New York, New York; ^3^ Department of Medicine and Division of Epidemiology, University of Alabama at Birmingham, Birmingham, Alabama.; ^4^ Department of Healthcare Policy and Research, Weill Cornell Medicine, New York, New York; ^5^ Division of Biostatistics and Epidemiology, Weill Cornell Medicine, New York, New York; ^6^ Department of Adult Reconstruction & Joint Replacement Service, Hospital for Special Surgery, New York, New York.; ^7^ Department of Orthopedic Surgery, Hospital of the University of Pennsylvania, Philadelphia, Pennsylvania.; ^8^ Department of Healthcare Delivery Science & Innovation, Weill Cornell Medicine, New York, New York

**Keywords:** race, rehabilitation, THA, readmissions

## Abstract

**Purpose:** Total hip arthroplasty (THA) is one of the fastest growing procedures. There is increasing evidence that social determinants of health influence health care utilization and outcomes after THA, including postoperative care. We sought to examine how race impacts discharge destination after elective THA, and we assessed the impact of discharge destination on 90-day readmission to an acute care hospital.

**Methods:** We conducted a retrospective study using data from the Pennsylvania Health Care Cost Containment Council Database. We included patients of African American (AA) or white race undergoing THA, discharge disposition (inpatient rehabilitation facility [IRF], skilled nursing facility [SNF], home health care (HHC), home), and 90-day readmission rates.

**Results:** Our study included 93,493 primary elective THAs. Compared with whites, AAs were more likely to be discharged to an IRF or SNF or HHC than home after THA. In all age groups, discharge to an IRF, SNF, or HHC for postop care/rehab was associated with higher odds of 90-day readmission as compared with home.

**Conclusion:** AA race was associated with higher odds of discharge to an institution (IRF/SNF) or HHC for post-THA care. Disposition to these were associated with significantly higher risk of 90-day readmission to acute care hospital compared with home.

## Introduction

Osteoarthritis (OA) is the most prevalent joint disease and a leading source of chronic pain and disability in the United States.^1^ Its prevalence has doubled even after controlling for many factors, including longevity and body mass index (BMI).^2^ Total hip arthroplasty (THA) is currently the only definitive treatment for patients with end-stage hip OA who have failed conservative treatment. THA has successful outcomes and a timely procedure can prevent functional disability in many patients, including the elderly. Elective THA is one of the fastest growing procedures and is the fourth most common surgical procedure performed surgical procedures in the United States.^3^

The utilization of THA has increased exponentially worldwide over the past decade and especially in United States where the rate exceeds 200/100,000 population.^4^ This utilization has also increased in the younger age group.^5^ Its utilization is anticipated to grow and by 2030, the demand for THA is expected to grow up to 572,000 THAs per year.^6^ In addition, there is growing evidence that social determinants of health, such as race, impact THA utilization and its outcomes. Even though the prevalence of Hip OA in African Americans (AAs) and whites is similar, AAs have lower utilization of THA.^7,8^

With regard to the outcomes, AAs have worse outcomes as compared with whites with a higher mortality, disability score, and longer length of stay.^9–11^ The rationale for these disparities are complex and intertwined between patient-level, provider-level, system-level, and policy-level factors. Utilization of postoperative care and its relationship to outcomes like readmissions is a lesser examined aspect in these disparities. We previously examined this in THA patients between the years 2002–2012 and found that race was a significant predictor of postoperative discharge destination. In addition, the discharge to an institution (inpatient rehabilitation facility [IRF] or skilled nursing facility [SNF]) was associated with increased odds of 90-day readmission to an acute care hospital.^12^

Since 2012, CMS has made several policy innovations to have an affordable and accessible health care system that makes patient outcomes a priority. Medicare Bundled Payment for Care Improvement (BPCI) initiative motivates and incentivize health care systems and providers to administer services efficiently and to better coordinate care, including discharge destination following THA. The hospitals are accountable for costs related to a THA procedure as well as costs associated within 90 days after discharge related to it.^13^ It is not known if these policy innovations had an impact on racial inequities in discharge disposition after THA. Therefore, using more recent data, we sought to examine the racial differences in discharge destination and if this is in turn associated with 90-day readmission to acute care hospital.

## Methods

### Data source

We retrospectively analyzed the Pennsylvania Health Care Cost Containment Council (PHC4) Database (2012–2016), which includes demographic data from all patient discharges from 170 nongovernmental acute care hospitals in the State of Pennsylvania. This includes all hospitals other than Veterans Administration or Military hospitals. The database was used to identify patients who underwent elective primary THA. Patients were identified using the International Classification of Diseases, Ninth Revision, Clinical Modification (ICD-9-CM) code 81.51 for primary THA from 2012 to September 2015, and ICD-10 codes 0SR90xx or 0SRB0xx thereafter. This study cohort and methodology has previously been described in detail.^14^

Our primary exposure of interest was race and thus only adults who identified as AAs or white and had an elective primary THA were included in this study. Study exclusion criteria included the following: patients with bilateral hip replacement, age <18, unknown race or insurance status, death on the same day of THA surgery or during hospitalization, transfer to a different acute care hospital or to a nonstudy destination (discharge destination other than the ones defined below), hip revision during the same hospitalization, or have more than two prior hip replacements (likely administrative dataset error).

### Outcome measures

The primary study outcome of interest was discharge disposition following a primary elective THA surgery. The discharge destination categories include IRF, SNF, home with home health care (HHC), and home with routine self-care (Home). In all analyses, discharge to home was used as the reference category. We also examined odds of 90-day readmissions to acute care hospital as our secondary outcome of interest.

### Analytical sample and covariates

We extracted information on important covariates, such as age, gender, insurance status (Commercial, Medicaid, Medicare, or other government-sponsored health insurance program), 90-day mortality, and clinical- and facility-level variables, from out dataset. We incorporated two facility-level variables related to the characteristics of the hospital at which the patients underwent THA. We used the 2013 U.S. Department of Agriculture's Rural/Urban Continuum Codes to assign the Metro area status to each hospital.^15^ The hospital THA procedure volume was categorized into three ordinal groups based on the volume: <100, 100–199, and ≥200 THA procedures per year. Complications, including postoperative myocardial infarction, prosthetic device complication, surgical wound infection, and venous thromboembolism, were identified using ICD-9/10 codes (Appendix Table A1). Lastly, medical comorbidities were identified using the Quan adaptation of the Elixhauser Comorbidity Index.^16,17^

For our secondary analysis, the primary predictor for 90-day readmission is the type of postsurgical rehabilitation care destinations (IRF, SNF, HHC, and home). The same covariates from the primary analysis were included in the multivariable logistic models for 90-day readmission. The study methods and results are described in accordance with the Strengthening of Reporting in Observational Studies in Epidemiology (STROBE) guideline for cohort studies.

### Statistical analyses

We compared the patient-level and facility-level characteristics, along with clinical outcomes, by patient race and stratified it by age group. We tested the associations between race and the various patient-level, facility-level, and outcome variables using Wald chi-square from unadjusted binary or multinomial logistic regression models, accounting for the clustering by Pennsylvania Area Facility. All models for the primary outcome considered race as the independent variable and accounted for clustering by hospital facility. Using similar strategies, patient-level characteristics, facility-level characteristics, and 90-day hospital readmission were compared by postsurgical discharge disposition (considered the independent variable).

We used multinomial logistic generalized linear mixed model^18^ to estimate the unadjusted and adjusted relative risk ratios (aRRRs) of being discharged to IRF, to a SNF, or to HHC (vs. home) after THA in AAs compared with whites. Multivariable models were adjusted for sex, age, insurance type, metro area, volume of cases, surgical complications (postoperative myocardial infarction, prosthetic device complication, surgical wound infection, venous thromboembolism) and Elixhauser Index. Covariates selection is based on clinical and prior knowledge from the scientific literature. In all models, patients were stratified by age group (<65 and 65 years or older). The age-based stratification was performed to account for differences in Medicare eligibility.

The relationship between 90-day readmission and discharge destination was assessed using binary logistic regression, accounting for clustering by hospital facility. Unadjusted and adjusted odds ratios (aORs) of hospital readmission at 90 days were estimated. Multivariable models were adjusted for the same patient-level and facility-level variables from the primary outcome analysis.

Furthermore, we utilized the coarsened exact matching (CEM) method in assessing the 90-day readmission outcome to reduce imbalance of covariates between different discharge destinations (IRF, SNF, HHC, and Home). CEM is a nonparametric and nonmodel-based matching method of the class monotonic imbalance bounding^19^ that ensures the balance of covariates between groups before estimation, allowing for less model dependence and reduced statistical bias than without matching.^20^ In CEM, adjusting imbalance in the empirical distribution in one covariate does not affect any other covariates chosen for balancing, which makes CEM more advantageous over other matching methods (King G, Nielsen R, Coberley C, et al. Comparative effectiveness of matching methods for causal inference. *Unpublished manuscript.* 2011;15:41). CEM was conducted using the CEM package in the 64-bit version of R.3.5.1.^21^ The dataset was matched across the following variables: age, gender, insurance, and 30 clinical comorbidities defined by Quan adaptation of Elixhauser comorbidities.^17^ After matching, 90-day readmission was regressed on discharge disposition using a binary logistic regression model adjusting for the same covariates in prior multivariable logistic regression.

Data management and analyses were conducted using SAS version 9.4 (SAS Institute, Cary, NC) and R.3.5.1 in RStudio (version 1.1.463; RStudio, Inc., Boston, MA).

## Results

### Study sample characteristics

**FIG. 1.**
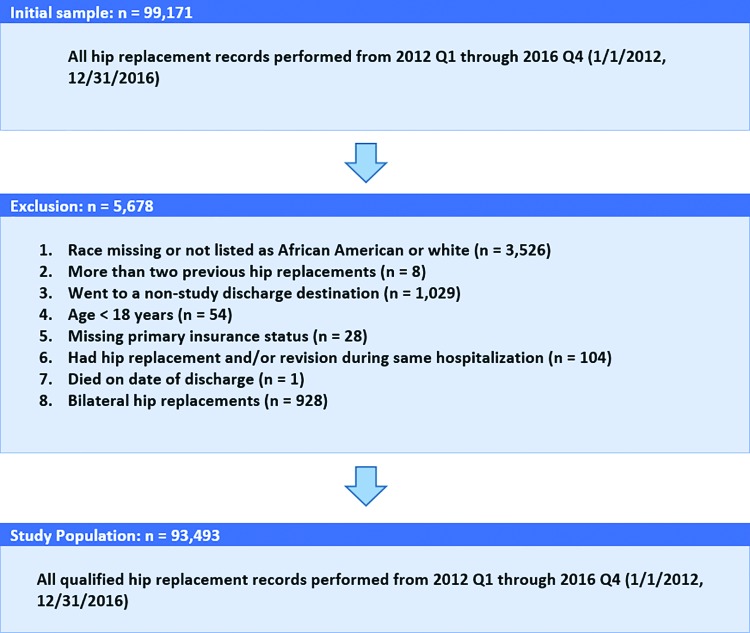
Sample flow chart and cohort selection.

Around 99,171 cases of THA were identified between 2012 and 2016 that met our inclusion criteria. We excluded 5678 cases for various reasons (Fig. 1). The final analytic sample was 93,493.

Baseline demographic and clinical characteristics of the cohort stratified by age group are summarized in Table 1. Briefly, among the THA surgery patients analyzed, 87,616 patients were white and 6626 were AA. In the age group less than 65 years (*n*=44,647), 9.5% were AA. Around 46.1% of AA and 47% of whites in that age group were female; 27% of AAs and 7.03% of whites relied on Medicaid insurance; whereas 80.3% whites and 50.4% AA relied on commercial insurance. In the age group 65 years and older (*n*=48,846), 4.5% were AA and rest white. Around 61.1% of whites and 65.5% of AAs in that age group were female; 0.14% of whites and 1.53% of AAs relied on Medicaid insurance; similar proportion of AAs and whites relied on commercial insurance in this older age group (∼12%).

**Table 1.** Baseline Characteristics and Outcomes by Race and Age Group (*N*=93,493)

Variable, *n* (%)	Years <65 (*n*=44,647)			Years ≥65 (*n*=48,846)		
	WH (*n*=40,394)	AA (*n*=4253)	*p*^a^	WH (*n*=46,619)	AA (*n*=2227)	*p*
Sex						
Female	18,977 (47.0)	1960 (46.1)		28,475 (61.1)	1458 (65.5)	^***^
Discharge facility type						
Home self-care	12,908 (32.0)	959 (22.5)	^***^	9281 (19.9)	385 (17.3)	^***^
HHC	13,441 (33.3)	1606 (37.8)		18,323 (39.3)	893 (40.1)	
SNF	5185 (12.8)	652 (15.3)		7008 (15.0)	357 (16.0)	
IRF	8860 (21.9)	1036 (24.4)		12,007 (25.8)	592 (26.6)	
Metro area						
Metro	37,794 (93.6)	4209 (99.0)		43,189 (92.6)	2201 (98.8)	
Nonmetro	2600 (6.44)	44 (1.03)		3430 (7.36)	26 (1.17)	
Insurance						
Unknown/uninsured	372 (0.92)	17 (0.40)	^***^	226 (0.48)	11 (0.49)	^***^
Medicare	4262 (10.6)	858 (20.2)		40,884 (87.7)	1909 (85.7)	
Medicaid	2838 (7.03)	1191 (28.0)		63 (0.14)	34 (1.53)	
Commercial	32,453 (80.3)	2142 (50.4)		5364 (11.5)	259 (11.6)	
Government	469 (1.16)	45 (1.06)		82 (0.18)	14 (0.63)	
Volume of cases (by facility and year)						
<100/year	5803 (14.4)	888 (20.9)		7438 (16.0)	424 (19.0)	
100–199/year	9621 (23.8)	641 (15.1)		12,235 (26.2)	333 (15.0)	
200+/year	24,970 (61.8)	2724 (64.0)		26,946 (57.8)	1470 (66.0)	
90-day readmission	3141 (7.78)	510 (12.0)	^***^	5198 (11.1)	292 (13.1)	
90-day mortality	50 (0.12)	13 (0.31)	^**^	312 (0.67)	8 (0.36)	
Elixhauser Index^b^						
0	7304 (18.1)	732 (17.2)		8006 (17.2)	410 (18.4)	
1–4	30,492 (75.5)	3264 (76.7)		35,726 (76.6)	1697 (76.2)	
≥5	2598 (6.43)	257 (6.04)		2887 (6.19)	120 (5.39)	
Surgery complications						
Myocardial infarction	22 (0.05)	1 (0.02)		126 (0.27)	5 (0.22)	
Prosthetic device complication	65 (0.16)	9 (0.21)		98 (0.21)	4 (0.18)	
Surgical wound infection	17 (0.04)	1 (0.02)		17 (0.04)	0 (0.00)	
Venous thromboembolism	11 (0.03)	2 (0.05)		64 (0.14)	3 (0.13)	

^a^ Variables are compared by race for each age group (years <65, ≥65) using Wald χ^2^ test from unadjusted binary or multinomial logistic regression models that account for clustering by facility. Significance levels: ^**^p<0.01, ^***^p<0.001.

^b^ Clinical comorbidities were identified based on coding algorithms developed by Quan et al. (enhanced Elixhauser version), using either the ICD-9-CM or the ICD-10 coding system, as appropriate. The Elixhauser comorbidity index score is calculated based on the cumulative number of comorbidity conditions.

AA, African American; HHC, home health care; IRF, inpatient rehabilitation facility; SNF, skilled nursing facility; WH, White.

### Characteristics by discharge destination: <65 years versus ≥65 years

Demographic and clinical characteristics by discharge destination were described (Table 2). Briefly, 38.2% of patients were discharged to either a SNF or an IRF, whereas the others were discharged to home self-care (Home) or HHC. Among AAs, 20.7%, 38.6%, 15.6%, and 25.1% were discharged to Home, HHC, SNF, and IRF, respectively. Among women, 22%, 38%, 14.7%, and 25.1% were discharged to Home, HHC, SNF, and IRF, respectively.

**Table 2.** Characteristics by Discharge Locations of Total Hip Replacement Recipients (*N*=93,493)

Variable, *n* (%)	Home self-care (*n*=23,533)	HHC (*n*=34,263)	SNF (*n*=13,202)	IRF (*n*=22,495)	*p*^a^
Race					
White	22,189 (94.3)	31,764 (92.7)	12,193 (92.4)	20,867 (92.8)	^***^
AA	1344 (5.71)	2499 (7.29)	1009 (7.64)	1628 (7.24)	
Sex					
Female	11,296 (48.0)	19,343 (56.5)	7480 (56.7)	12,751 (56.7)	^***^
Male	12,237 (52.0)	14,920 (43.5)	5722 (43.3)	9744 (43.3)	
Age group					
Years <65	13,867 (58.9)	15,047 (43.9)	5837 (44.2)	9896 (44.0)	^***^
Years ≥65	9666 (41.1)	19216 (56.1)	7365 (55.8)	12,599 (56.0)	
Metro area					
Metro	21,124 (89.8)	32,458 (94.7)	12,507 (94.7)	21,304 (94.7)	
Nonmetro	2409 (10.2)	1805 (5.27)	695 (5.26)	1191 (5.29)	
Insurance					
Unknown/uninsured	225 (0.96)	207 (0.60)	76 (0.58)	118 (0.52)	^***^
Medicare	8961 (38.1)	19,092 (55.7)	7324 (55.5)	12,536 (55.7)	
Medicaid	926 (3.93)	1575 (4.60)	629 (4.76)	996 (4.43)	
Commercial	13,223 (56.2)	13,203 (38.5)	5085 (38.5)	8707 (38.7)	
Government	198 (0.84)	186 (0.54)	88 (0.67)	138 (0.61)	
Volume of cases (by facility and year)					
<100/year	2231 (9.48)	5976 (17.4)	2378 (18.0)	3968 (17.6)	
100–199/year	4155 (17.7)	9254 (27.0)	3313 (25.1)	6108 (27.2)	
200+/year	17,147 (72.9)	19,033 (55.5)	7511 (56.9)	12,419 (55.2)	
90-day readmission	1476 (6.27)	3766 (11.0)	1424 (10.8)	2475 (11.0)	^***^
90-day mortality	25 (0.11)	184 (0.54)	63 (0.48)	111 (0.49)	^***^
Elixhauser Index^b^					
0	4203 (17.9)	6006 (17.5)	2317 (17.6)	3926 (17.5)	
1–4	17,852 (75.9)	26,096 (76.2)	10,061 (76.2)	17,170 (76.3)	
≥5	1478 (6.28)	2161 (6.31)	824 (6.24)	1399 (6.22)	
Surgery complications					
Myocardial infarction	12 (0.05)	69 (0.20)	28 (0.21)	45 (0.20)	^***^
Prosthetic device complication	21 (0.09)	81 (0.24)	28 (0.21)	46 (0.20)	^***^
Surgical wound infection	7 (0.03)	18 (0.05)	5 (0.04)	5 (0.02)	
Venous thromboembolism	3 (0.01)	48 (0.14)	14 (0.11)	15 (0.07)	^***^

^a^ Variables are compared by discharge destination using Wald χ^2^ test from unadjusted binary or multinomial logistic regression models that account for clustering by facility. Significance levels: ^***^p<0.001.

^b^ Clinical comorbidities were identified based on coding algorithms developed by Quan et al. (enhanced Elixhauser version), using either the ICD-9-CM or the ICD-10 coding system, as appropriate. The Elixhauser comorbidity index score is calculated based on the cumulative number of comorbidity conditions.

Among those with Medicare, 18.7% were discharged to home and 41.5% and 37.5% were discharged to SNF or IRF, respectively. Thus, a higher percentage of patients were discharged to an institution when patients had Medicare. Among those with commercial insurance, 32.9% were discharged to home and 34.2% were discharged to SNF or IRF, showing that a higher proportion was discharged home when they had commercial insurance.

### Race and discharge destination

**FIG. 2.**
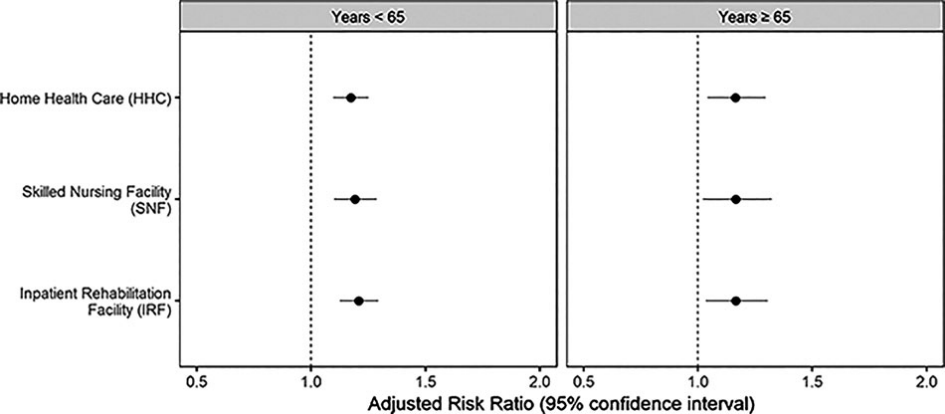
Adjusted relative risk ratios of referral to varying discharge locations in African American THA patients (vs. whites) in two age groups. The generalized multinomial logit model accounts for clustering by facility. Adjusted covariates included patient sex, insurance, facility type, facility volume of cases, Elixhauser Index, and surgical complications (venous thromboembolism, prosthetic device complications, myocardial infarction, and wound infections). THA, total hip arthroplasty.

We estimated relative risk ratios of discharge destination by patient race, adjusting for important patient- and facility-level confounders (Fig. 2). Among patients younger than 65 years, compared with whites, AAs were more likely to be discharged to IRF (aRRR: 1.21, 95% confidence interval [CI]: 1.13–1.29), SNF (aRRR: 1.19, 95% CI: 1.10–1.29) or HHC (aRRR: 1.17, 95% CI: 1.10–1.25), but not home. Similarly, in the older age group >65, compared with whites, AAs were more likely to be discharged to an IRF (aRRR: 1.16, 95% CI: 1.03–1.31), SNF (aRRR: 1.17, 95% CI: 1.02–1.32), or HHC (aRRR: 1.16, 95% CI: 1.04–1.30) compared with Home.

### Discharge disposition and 90-day readmission to acute-care hospital

**FIG. 3.**
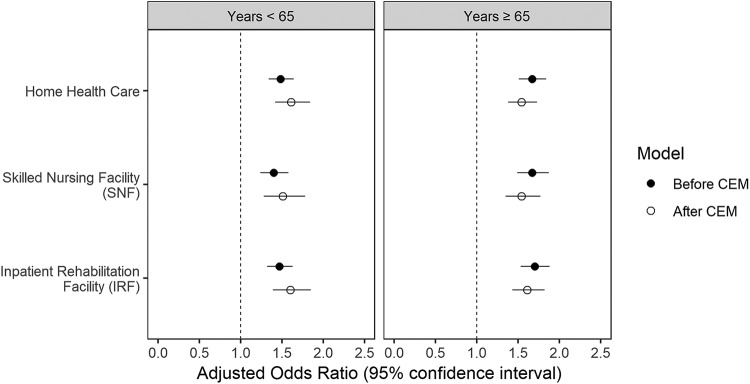
Adjusted odds ratios of 90-day readmission in patients who were discharged to various locations compared with home before and after CEM. The binary logistic 90-day readmission models adjusted for patient sex, race, insurance, Elixhauser Index, facility type, facility volume of cases, and surgical complications. CEM, coarsened exact matching.

The association of discharge disposition with 90-day readmission to an acute care hospital was analyzed after adjusting for confounders as well as after CEM (Fig. 3). In the younger age group (less than 65 years) compared with Home, discharge to IRF, SNF, and HHC was associated with higher odds of 90-day readmission to acute care hospital (aOR: 1.47, 95% CI: 1.32–1.63; aOR: 1.40, 95% CI: 1.24–1.58; and aOR: 1.48, 95% CI: 1.34–1.64, respectively). This association remained statistically significant (*p*<0.05) after CEM for IRF, SNF, and HHC (aOR: 1.60, 95% CI: 1.39–1.85; aOR: 1.51, 95% CI: 1.28–1.78; and aOR: 1.61, 95% CI: 1.42–1.84, respectively) compared with Home.

Similarly, in the older age group, compared with Home, discharge to IRF, SNF, or HHC, was associated with higher odds of 90-day readmission to acute care hospital (aOR: 1.70, 95% CI: 1.53–1.88; aOR: 1.67, 95% CI: 1.49–1.87; and aOR: 1.67, 95% CI: 1.51–1.84, respectively). This association remained statistically significant after we repeated the analysis using CEM. The adjusted odds for IRF, SNF, and HHC were aOR: 1.61, 95% CI: 1.43–1.82; aOR: 1.54, 95% CI: 1.35–1.77; and aOR: 1.54, 95% CI: 1.38–1.73, respectively.

## Discussion

In this study of a large-scale regional database, we observed that there were significant differences between racial groups in postoperative discharge destination after elective THA. In this statewide dataset, when compared with whites, AAs were significantly more likely discharged to an Institution (IRFs or SNFs) or have HHC for postoperative care rather than home. We found that these differences persisted even after controlling for individual-level demographics, comorbidities, postoperative complications as well as facility-level characteristics. Furthermore, compared with home, discharge to IRF, SNF, and HHC was associated with higher odds of 90-day admission rate, even after adjusting for confounders and methodically matching of postop complications and comorbidities.

Earlier studies examining discharge destination after THA did not test the association of race. For example, in a multicenter study of patients from patients in Mayo clinic, Rochester; Massachusetts General Hospital, Boston, and University of California, San Francisco (4485 patients), 29% were discharged to inpatient extended care facilities. Older age >80 or greater, female gender, higher comorbidities (American Society of Anesthesiologists class), and Medicare insurance were most significant predicators of discharge to an inpatient facility, however, patient race was not reported in this study.^22^ However, our findings are similar to the previous studies that have examined race.^23,24^ In a single institutional administrative data study, minorities were more likely to be assigned to an institution after discharge (OR: 2.11).^25^ In a larger study using California statewide data (*n*=14,326), race, insurance, and morbidity were the main driving factors on patient discharge to SNF.^26^

With regard to readmission, a single-center study from New York using hospital records from 2010 to 2011 demonstrated that patients discharged home with health services has significantly lower readmission rates than those discharged to IRFs (1.5% vs. 5.1%).^27^ A study from Kaiser Permanente total joint replacement registry from 2001 to 2004 (n_THA_=3432), after controlling for the comorbidities, age, sex, and hospital complications, the records of hospital readmission were higher in patients discharge to an SNF.^28^ Finally, in a previous study by our team analyzing the PHC4 database from 2002 to 2012, similarly demonstrated that when compared with whites, AA patients had significantly higher odds of discharge to IRF (age <65, aRRR=2.56; age ≥65, aRRR=1.96) and to SNF (age <65, aRRR=3.37; age ≥65, aRRR=3.66). In addition, the OR of 90-day readmission was higher in patients who were discharged to IRF or SNF irrespective of the age group (age <65, OR_IRF_ 4.06, OR_SNF_ 2.05; age ≥65, OR_IRF_ 4.32, OR_SNF_ 1.74).^12^

Our results demonstrating that AA patients are more likely than white patients to be discharged to an institution (IRF/SNF) or HHC for postoperative care following elective THA are important. We demonstrated that even after the change in CMS policies, marked disparities remain in our analysis from 2012 to 2016. However, it is important to note that the aRRRs have improved for AAs to be discharged to a nonhome disposition. In addition, the ORs for 90-day readmission to an acute care hospital have decreased. The CEM method confirmed the findings of the adjusted model. However, patients <65 years had a stronger predisposition, whereas patients >65 years had a weaker predisposition to 90-day readmission after CEM. Thus, describing that the matching made a difference in effect sizes, however, the association remained significant. Studies have shown that early discharge to home is associated with reduced cost, improved clinical outcomes, and increased patient satisfaction.^29,30^ Thus, our study findings are significant since elective THA is one of the most important cost centers for insurances and postoperative care contributed a significant amount to the costs.^29–31^ Also, discharge to an institution is associated with higher odds of readmission rates, which is a key outcome measure post THA.^32^ It is possible that the discharge destination is not only determined by clinical parameters but also some social determinants like proximity to home, social and community support, or overall deprivation or social vulnerability, of which race may be a marker.^32,33^

Our study has several limitations. This large administrative database does not contain information on potentially important confounding variables such as BMI, social support or patient preference in disposition, or regarding patient-reported outcomes, and we are unable to comment on these factors. Several patients may have transferred between different facilities during postoperative care and it is difficult to capture these adequately and thus, the crossover effect cannot be estimated in our study. We studied only patients with primary elective THA in the Pennsylvania, thus, our results may not be generalizable to other regions or states of the country, because there is a significant geographical variation in several key factors, including social determinants of health.^34^

## Conclusion

In summary, this large-scale study of 99,171 patients undergoing primary THA across 170 Pennsylvania hospitals demonstrated that as compared with whites, AA patients were more likely to be referred to institution (IRFs and SNFs) or HHC for postoperative care rather than home. Moreover, discharge to an institution or HHC for postoperative care increases the odds of 90-day hospital readmission when compared with being discharged home. Evaluations of the decision process regarding discharge destination for postacute care and rehabilitation following elective THA are needed. We also need studies to examine how social determinants of health such as patient race impact these decisions.

## Abbreviations Used

AA: African American
aRRR: adjusted relative risk ratio
BMI: body mass index
BPCI: Bundled Payment for Care Improvement
CEM: coarsened exact matching
HHC: home health care
IRF: inpatient rehabilitation facility
SNF: skilled nursing facility
STROBE: Strengthening of Reporting in Observational Studies in Epidemiology
THA: total hip arthroplasty

## Appendix

**Appendix Table A1.** ICD-9/10 Codes of Inclusion Hip Procedure and Surgical Complications

	ICD 9 codes	ICD 10 codes
Hip replacement	8151	0SR90xx, 0SRB0xx
Venous thromboembolism	45340, 45341, 4536, 45342, 45384, 45381, 45386, 45389, 45382, 4539, 45385	I82441, I82433, I82B19, I82409, I82621, I82411, I82402, I82401, I824Z9, I82491, I824Z1, I82432, I82412, I824Z2, I82512
Acute myocardial infarction	41071, 41021, 41011, 41072, 41081, 41001, 41061, 41091, 41042, 41070, 41090, 41020, 41041	I214, I213, I2129, I2119, I2102
Surgical wound infection	99859	K6811, T814XXA
Prosthetic device complications	99677, 99647, 99642, 99678, 99644, 99640, 99649, 99643, 99666, 99667, 99641, 99601, 99646, 99679	T8481XA, T84115D, T84020A,T84041A, T84114A, T84218A, T8484XA, T84498A, T84199A, T84011A, T84021A, T8489XA, T8451XA, T84010A, T84091A, T84031A, T84328A, T84030A, T847XXA, T84050S, T84099A, T84040A, T84090A, T84428A, T8451XD, T84038A, T84061A, T84050A, T8486XA, T8452XA, T8483XA
